# Anti‐Müllerian hormone and vascular dysfunction in women with chronic kidney disease

**DOI:** 10.14814/phy2.15154

**Published:** 2022-01-25

**Authors:** Sandra M. Dumanski, Todd J. Anderson, Kara A. Nerenberg, Jayna Holroyd‐Leduc, Jennifer MacRae, Satish R. Raj, Amy Metcalfe, Sharanya Ramesh, Cindy Z. Kalenga, Darlene Sola, Milada Pajevic, Sofia B. Ahmed

**Affiliations:** ^1^ Department of Medicine University of Calgary Calgary Alberta Canada; ^2^ Libin Cardiovascular Institute of Alberta Calgary Alberta Canada; ^3^ Alberta Kidney Disease Network Calgary Alberta Canada; ^4^ Department of Community Health Sciences University of Calgary Calgary Alberta Canada; ^5^ Department of Obstetrics and Gynecology Foothills Medical Center University of Calgary Calgary Alberta Canada; ^6^ Department of Cardiac Sciences University of Calgary Calgary Alberta Canada; ^7^ Faculty of Medicine University of Toronto 1 King’s College Circle Toronto Ontario Canada; ^8^ Cumming School of Medicine University of Calgary Calgary Alberta Canada

**Keywords:** arterial stiffness, cardiovascular, endothelial dysfunction, fertility, ovarian function

## Abstract

Young women with chronic kidney disease (CKD) have disproportionately increased risk of cardiovascular mortality. Reduced anti‐Müllerian hormone (AMH) is linked to poor cardiovascular outcomes in the general population, but whether AMH is associated with increased cardiovascular risk in the high‐risk CKD population is unknown. This study examined the association between AMH and vascular function, validated markers of cardiovascular risk, in women with CKD. An exploratory cross‐sectional study was performed in 47 young women with CKD. Laboratory measurements of AMH were collected. Using standardized protocols, endothelial function was measured with brachial artery flow‐mediated dilation and hyperemic velocity time integral. Arterial stiffness was measured with aortic augmentation index and pulse wave velocity. Multivariate linear regression analyses were utilized to evaluate the association between AMH levels and each measure of vascular health. Forty women (36 ± 7 years) with non‐dialysis‐dependent CKD and 7 women (38 ± 6 years) with dialysis‐dependent CKD participated. AMH levels were inversely associated with age (*p* = 0.01) but not associated with eGFR (*p* = 0.59) or dialysis status (*p* = 0.97). AMH was associated with brachial artery flow‐mediated dilation (*R* ^2^ = 0.21 [*p* = 0.03]) and aortic augmentation index (*R* ^2^ = 0.20 [*p* = 0.04]) in the non‐dialysis‐dependent participants, and with aortic augmentation index in all participants (*R* ^2^ = 0.18 [*p* = 0.03]). No association between AMH and any measure of vascular function was demonstrated in the dialysis‐dependent participants. AMH levels are associated with impaired vascular function in young women with CKD and may be an important marker of future cardiovascular risk. Further investigation into this female‐specific cardiovascular risk factor is warranted in this high‐risk population.

## 1 INTRODUCTION

On a global scale, cardiovascular disease (CVD) is the leading cause of female mortality, accounting for one in three deaths in women (GBD, 2017 Causes of Death Collaborators, 2018). Chronic kidney disease (CKD) is an important risk factor for CVD and cardiovascular mortality, and compared to the general population, those with CKD have up to 30 times increased risk of CVD‐related death (GBD, 2017 Causes of Death Collaborators, 2018; Sarnak et al., 2002). CKD is defined by a reduced estimated glomerular filtration rate (eGFR) and/or albuminuria, and each of these markers are independently associated with an increased CVD risk (Chronic Kidney Disease Consortium, 2010; GBD, 2017 Causes of Death Collaborators, 2018; Hemmelgarn et al., 2010). As a CVD risk factor, CKD is particularly important in women, as the CVD‐related mortality risk relationship with both eGFR and albuminuria is stronger in women compared to men (Nitsch et al., 2013). Furthermore, significantly increased cardiovascular mortality is demonstrated in women with earlier stages of kidney disease (eGFR >75 ml/min/1.73 m^2^), but not in men (Nitsch et al., 2013).

In young premenopausal‐aged women, CKD is a distinctly important mortality risk factor. Unlike the population of postmenopausal women with CKD, and in stark contrast to the general population, young women with CKD have an *increased* all‐cause mortality compared to age‐matched men with CKD, and do not demonstrate an overall sex‐related survival advantage in terms of CVD mortality (Carrero et al., 2011). Despite implementation of advancing CVD treatments over the last three decades, women have continued to experience excess cardiovascular mortality risk compared to men, especially pronounced in the young female demographic (<55 years), who have experienced minimal reduction in CVD‐related mortality alongside increasing CVD hospitalization rates during this time (Arora et al., 2019; Izadnegahdar et al., 2014; Wilmot et al., 2015). Understanding novel non‐traditional CVD disease mechanisms and risk factors specific to young women, especially in the high‐risk population of young women with CKD, is needed to inform clinical practice and advance sex‐specific CVD care for this vulnerable population (Yinon et al., 2010).

Female factor infertility and female sex hormones have been linked to increased risk of CVD and poorer cardiovascular outcomes in young women (Hanson et al., 2017; Mahalingaiah et al., 2017; Moreau et al., 2013; Quinn & Cedars, 2018). Recent data have demonstrated that reduced anti‐Müllerian hormone (AMH), a surrogate marker of ovarian reserve that declines alongside the follicular reserve during the female reproductive life span (Jirge, 2011), is independently associated with CVD in young women (Appt et al., 2012; de Kat et al., 2016, 2017; Kim et al., 2017; Looby et al., 2016; Shand et al., 2014; Tokmak et al., 2015; Yarde et al., 2014). CKD is associated with a notable reduction in fertility compared to the general population, and women with CKD have significantly lower levels of AMH (Sikora‐Grabka et al., 2016; Stoumpos et al., 2018; Wiles et al., 2021), but whether AMH levels are associated with CVD risk in young women with CKD is yet unknown.

The aim of this study was to determine the association between AMH and measures of vascular function in young women with CKD, including endothelial function and arterial stiffness, validated measures of cardiovascular risk (Martin & Anderson, 2009; Vlachopoulos et al., 2010). We hypothesized that reduced AMH would be associated with decreased vascular function in young women with CKD.

## 2 MATERIALS AND METHODS

### 2.1 Study population

Self‐identified women between the ages 18 and 50 years with either non‐dialysis‐ or dialysis‐dependent CKD were invited to participate in the study. Participants were recruited from Alberta Kidney Care South nephrology clinics in Calgary, Alberta, and received written permission to participate in the study from their primary kidney care provider. Exclusion criteria included current assisted reproductive therapy, pregnancy, or breastfeeding. Participants with a history of polycystic ovarian syndrome, premature ovarian insufficiency, ovarian malignancy, gonadotoxic chemotherapy, surgical oophorectomy or hysterectomy, surgical management of ovarian cysts/masses or endometriosis, or previous radiation to the pelvis were also excluded.

### 2.2 Study protocol

The study protocol was approved by the Conjoint Health Research Ethics Board at the University of Calgary (REB18‐0642), and was conducted in accordance with institutional policy. Each study participant provided written informed consent to participate in the study. Study participants underwent a 12‐h fast and abstained from alcohol, smoking, and caffeine for 4 h prior to study appointment. During this time, only prescribed medications were taken with sips of water. Women with dialysis‐dependent CKD treated with hemodialysis were studied on the day after a hemodialysis session. All menstruating participants were studied in the follicular phase of their menstrual cycle (between day 1 and day 14 of their menstrual cycle). Participants were examined in the supine position in a quiet, temperature‐controlled room. Study measurements were taken after 10 min of rest without speaking or sleeping. All measurements and data collection were completed in a single laboratory session by two trained research assistants (a senior study nurse responsible for all baseline medical history collection, physical examination, laboratory collection, and measures of arterial stiffness; an experienced research assistant responsible for measurements of endothelial function). Demographic and medical history were collected, including a detailed kidney disease history, a history of cardiovascular comorbidities, menstrual and reproductive history, and medication history. A physical examination included anthropomorphic measurements (height, weight, and body mass index [BMI]), as well as heart rate and blood pressure measured according to Hypertension Canada guidelines (Nerenberg et al., 2018). Baseline laboratory characteristics included creatinine, eGFR (using the CKD‐EPI equation), and urine albumin‐to‐creatinine ratio (ACR).

### 2.3 Measure of exposure

The exposure was defined as the participant's serum level of AMH from a single reading measured the day of the study. AMH levels were determined using a validated immunoassay (Elecsys^®^ AMH, Roche Diagnostics) on an automated system (Cobas e601, Roche Diagnostics) (Klemt et al., 2020). The assay has a known imprecision of <3.5% and was calibrated and quality controlled using the manufacturer's reagents. The measuring range of the AMH test is 0.071–164.2 pmol/L, which includes all values in the normal reference range for premenopausal women (0.3–67.8 pmol/L). A centralized laboratory (Alberta Precision Laboratories) performed all study tests.

### 2.4 Outcome measures

Using a previously published protocol (Martin et al., 2013), conduit vessel endothelial function was measured on each participant. Right brachial artery flow‐mediated dilation (FMD) was measured utilizing ultrasound imaging (Phillips). Two‐dimensional images of the brachial artery were captured at baseline to obtain arterial diameter, as well as blood flow velocity using Doppler. Following baseline imaging assessment, a shear stress stimulus (in the form of a sphygmomanometer) was applied to the participant's right arm at a suprasystolic pressure (50 mmHg above systolic blood pressure [SBP]) for 5 min. Following deflation of the cuff, reactive hyperemia was induced and the brachial artery dilated in response to nitric oxide, with maximal dilation occurring typically between 45 and 120 s. Ultrasound imaging of the arterial dilation and tracking of the blood flow velocity was completed for 120 s following cuff deflation on each participant. FMD was reported as a percent change in arterial diameter from baseline (%). During the same procedure for FMD as described above, microvascular arterial dilation during cuff occlusion resulted in increased macrovascular blood flow in the reactive hyperemia period. Velocity time integral (VTI) is a measurement of the blood flow distance for a specified time period, and was calculated automatically using ultrasound Doppler images of the first beat of reactive hyperemia mapped on a velocity–time graph and measured in centimeters (cm). All endothelial function analyses were completed with Brachial Analyzer for Research version 5 (Medical Imaging Applications).

Using a standardized protocol (Abdi‐Ali et al., 2014), arterial stiffness was measured with the aortic augmentation index (AIx) and pulse wave velocity (PWV) through non‐invasive applanation tonometry (Millar Instruments). To measure PWV, the velocity of each participant's arterial pulse wave was measured by recording a pulse profile with an arterial tonometer at two distinct locations (carotid and femoral arteries). The time the pulse took to transmit (Δ*t*), as well as the transmitted distance (Δ*D*), was measured through graphical capture of the pulsatile waveform and standardized body surface measurement, respectively. PWV was calculated as Δ*D*/Δ*t* (m/s). Augmentation of the aortic pulse, or AIx, is measured using pulse wave analysis, and calculated by the rise or fall from the initial shoulder of the waveform in early systole, to the late systolic shoulder of the waveform. AIx was expressed in percentage (%). All arterial stiffness analyses were completed utilizing SphygmoCor version 8 (AtCor Medical).

### 2.5 Statistical analysis

Baseline descriptive and categorical data were stratified by dialysis status and were summarized as mean ± standard deviation (SD) and percentages, respectively. Continuous variables between the two groups were compared using the Mann–Whitney *U* test, and categorical variables were compared utilizing the chi‐squared test. The associations between AMH and each primary outcome measurement (FMD, VTI, AIx, and PWV) were examined using univariate and multivariate linear regression analyses. In addition to AMH, relevant covariates (age, eGFR, ACR, and menstruation) and interactions were included in the analysis, and stepwise backward elimination was utilized to determine a final model. Statistical analyses were performed using STATA version 15.0 (StataCorp), and were two‐tailed with a significance level of 0.05.

## 3 RESULTS

### 3.1 Baseline characteristics

Participant characteristics are presented in Table 1. Participants were predominantly Caucasian (57%). The majority of participants were normotensive with a normal resting heart rate, and had pre‐existing cardiovascular comorbidities, the most common being hypertension. More than one third of participants were on angiotensin‐converting enzyme (ACE) inhibitors or angiotensin II receptor blockers (ARBs), but other cardiovascular medications were infrequent. The non‐dialysis‐dependent CKD group had significantly higher BMI than the dialysis‐dependent CKD group (*p* = 0.04). The most common CKD etiology in both groups was glomerular disease (34% IgA nephropathy, 21% lupus nephritis, 8% membranoproliferative glomerulonephritis, 8% focal segmental glomerulosclerosis, and 29% unknown/other). The non‐dialysis‐dependent CKD group exhibited a broad range of eGFR (13–138 ml/min/1.73 m^2^) and albuminuria measurements (ACR 0.3–806.4 mg/mmol), with the majority of participants in early CKD stages with minimal urinary albumin excretion. Women with non‐dialysis‐dependent CKD reported a shorter duration of CKD compared to women with dialysis‐dependent CKD, but this difference was not significant (*p* = 0.47). The majority of women with dialysis‐dependent CKD were treated with hemodialysis (86%). Amenorrhea was reported in approximately one third of all participants, and rates of menstruation and regular menstruation were higher in the non‐dialysis‐dependent CKD group, but these differences did not reach statistical significance (*p* = 0.60 and 0.24, respectively).

**TABLE 1:** Baseline characteristics

	Non‐dialysis‐dependent CKD	Dialysis‐dependent CKD
*N*	40	7
Age (years)	36 ± 7	38 ± 6
Self‐identified ethnicity		
Caucasian	60%	43%
South East Asian	15%	43%
South Asian	8%	0%
Indigenous	8%	0%
East Asian	5%	0%
Latina	2%	14%
Black	2%	0%
BMI (kg/m^2^)	28.6 ± 7.7	22.9 ± 5.9*
SBP (mmHg)	125 ± 12	133 ± 27
DBP (mmHg)	75 ± 9	74 ± 17
HR (BPM)	60 ± 10	67 ± 10
Cardiovascular comorbidities		
Hypertension	58%	71%
Dyslipidemia	23%	29%
Diabetes	18%	14%
Stroke/TIA	3%	14%
CAD/ACS	3%	0%
Cardiovascular medications		
ACE inhibitor or ARB	40%	29%
ß blocker	3%	29%
Calcium channel blocker	3%	29%
Diuretic	13%	0%
Type of dialysis	N/A	
Hemodialysis		86%
Peritoneal dialysis		14%
CKD stage		
GFR category		N/A
G1 (eGFR ≥90)	55%	
G2 (eGFR 60–89)	25%	
G3 (eGFR 30–59)	17%	
G4 (eGFR 15–29)	0%	
G5 (eGFR <15)	3%	
Albuminuria category		
A1 (ACR <3)	65%	
A2 (ACR 3–30)	15%	
A3 (ACR >30)	20%	
eGFR (ml/min/1.73 m^2^)	88 ± 29	N/A
ACR (mg/mmol)	39 ± 130	64 ± 63
Anuria	0%	71%*
Duration of CKD (years)	9.3 ± 9.3	11.9 ± 10.6
Cause of CKD		
Glomerular disease	45%	86%
Polycystic kidney disease	15%	0%
Medullary sponge kidney ± nephrolithiasis	15%	0%
Diabetes	10%	14%
Reflux nephropathy ± obstructive nephropathy	8%	0%
Hypertension	5%	0%
Drug‐induced kidney disease	2%	0%
Menstrual health		
Reports menstruation	68%	57%
Regular menses^a^, ^b^	78%	50%
On menses‐suppressing contraception^c^	7%	0%
Estradiol (pmol/L)	305 ± 368	600 ± 661
Progesterone (nmol/L)	2.7 ± 4.7	1.0 ± 0.5
AMH (pmol/L)	15.4 ± 15.1	15.5 ± 11.3

Note

Data are mean ± SD, unless otherwise indicated.

Abbreviations: ACE, angiotensin‐converting enzyme; ACR, albumin creatinine ratio; ACS, acute coronary syndrome; AMH, anti‐Mullerian hormone; ARB, angiotensin II receptor blocker; BMI, body mass index; CAD, coronary artery disease; CKD, chronic kidney disease; DBP, diastolic blood pressure; eGFR, estimated glomerular filtration rate; HR, heart rate; SBP, systolic blood pressure; TIA, transient ischemic attack.

^a^ Menses every 24–38 days.

^b^ Of those reporting menstruation.

^c^ Injectable contraception.

* *p* < 0.05 versus non‐dialysis‐dependent CKD.

### 3.2 Baseline anti‐Müllerian hormone and vascular health measurements

**FIGURE 1:**
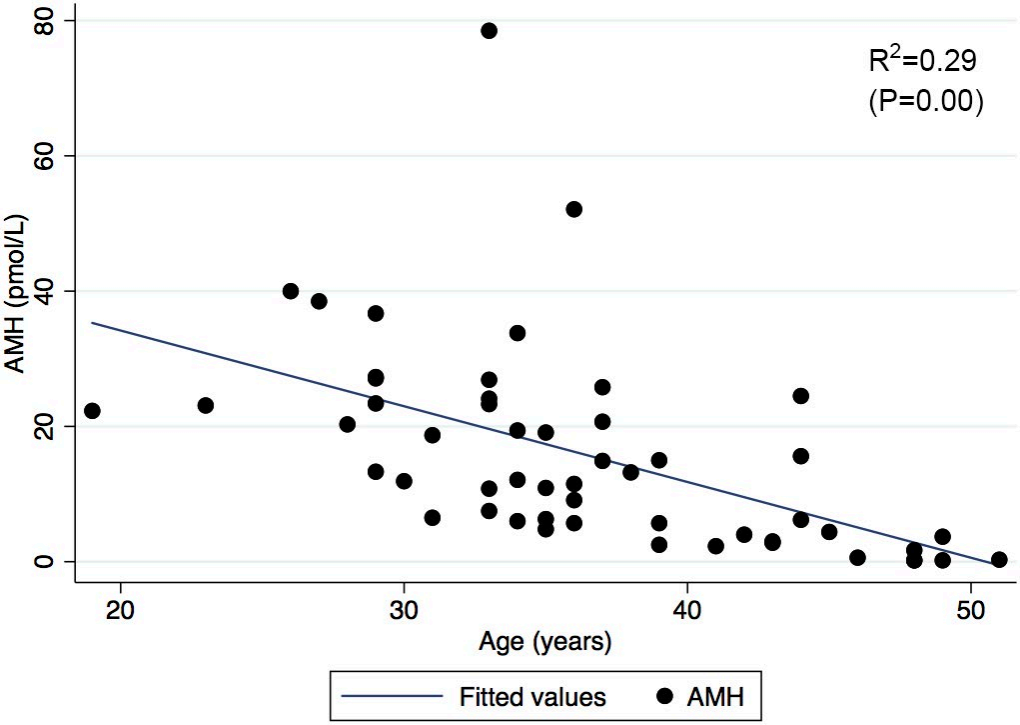
Relationship between AMH level and age in women with CKD. A strong association was demonstrated between AMH and age in study participants, with younger‐aged women demonstrating higher levels of AMH, a similar pattern to the general population. AMH, anti‐Mullerian hormone; CKD, chronic kidney disease

**FIGURE 2:**
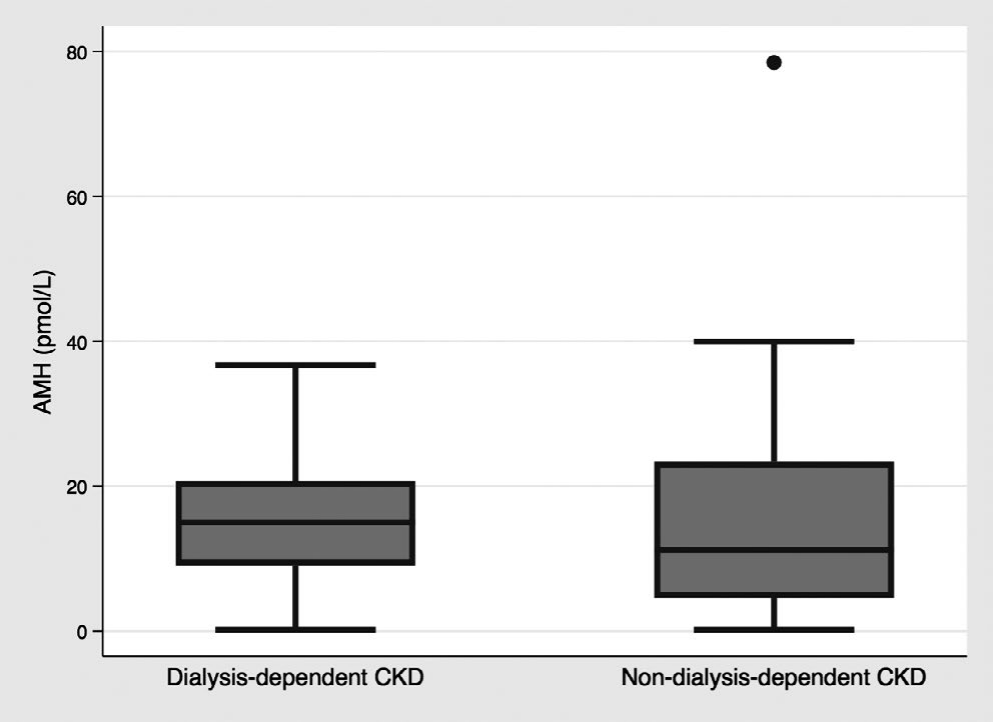
Anti‐Mullerian hormone levels in women with dialysis‐dependent CKD and non‐dialysis‐dependent CKD. No difference in AMH levels between the two groups was exhibited, with similar AMH levels reported in the dialysis‐dependent and non‐dialysis‐dependent CKD groups. *p < 0.05. AMH, anti‐Mullerian hormone; CKD, chronic kidney disease

**FIGURE 3:**
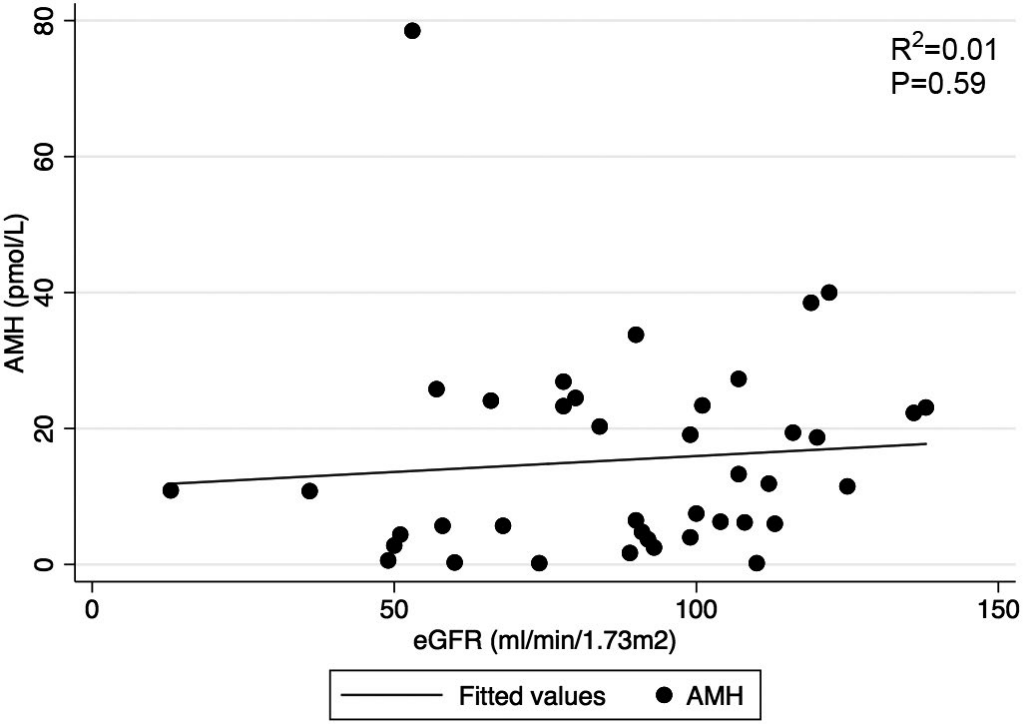
Relationship between AMH level and eGFR in women with non‐dialysis‐dependent CKD. There was no significant association illustrated between AMH and eGFR in the non‐dialysis‐dependent study group, which suggests that AMH level may not decline alongside progression of CKD. AMH, anti‐Mullerian hormone; CKD, chronic kidney disease; eGFR, estimated glomerular filtration rate

**FIGURE 4:**
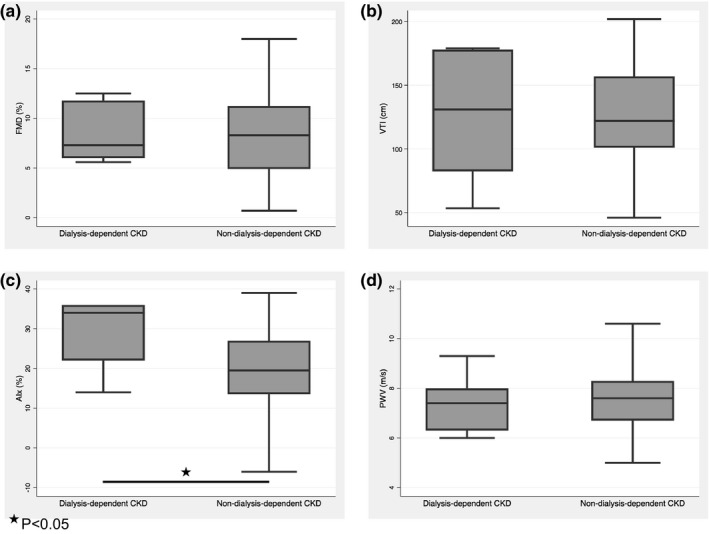
Comparison of vascular health parameters in women with non‐dialysis‐dependent CKD and dialysis‐dependent CKD. Although baseline endothelial function measurements (FMD & VTI) and pulse wave velocity (PWV) did not differ between the non‐dialysis‐dependent and dialysis‐dependent CKD groups (panel a, b, d, respectively), the dialysis‐dependent CKD group demonstrated increased AIx, a measure of arterial stiffness, as compared to the non‐dialysis‐dependent CKD group. *p < 0.05. AIx, aortic augmentation index; CKD, chronic kidney disease; FMD, flow‐mediated dilation; PWV, pulse wave velocity; VTI, velocity time integral

Participants demonstrated a wide range of AMH levels (0.2–78.5 pmol/L), and a strong association was demonstrated between AMH and age (Figure 1). There was no statistically significant difference in AMH level between participants when stratified by dialysis status (*p* = 0.74) (Figure 2). Furthermore, AMH level had no significant association with eGFR (Figure 3), and did not differ between the CKD stages (*p* = 0.46). Baseline vascular health measurements are presented in Table 2. When stratified by dialysis status, the participants did not differ in terms of baseline endothelial function measurements (A,B) (Figure 4). Participants with dialysis‐dependent CKD had significantly higher AIx compared to participants with non‐dialysis‐dependent CKD (*p* = 0.03) (C), but no difference in PWV was observed (D). No significant differences were demonstrated in FMD, VTI, AIx, or PWV, when stratified by CKD stage (*p* = 0.42, 0.33, 0.79, 0.16, respectively).

**TABLE 2:** Measures of vascular health in women with non‐dialysis‐dependent CKD and dialysis‐dependent CKD

	Non‐dialysis‐dependent CKD	Dialysis‐dependent CKD
AIx (%)	19 ± 12	30 ± 9*
PWV (m/s)	7.5 ± 1.2	7.4 ± 1.2
FMD (%)	8 ± 4	8 ± 3
VTI (cm)	126 ± 37	124 ± 51

Note

Data are presented as mean ± SD.

Abbreviations: Aix, aortic augmentation index; CKD, chronic kidney disease; FMD, flow‐mediated dilation; PWV, pulse wave velocity; VTI, velocity time integral.

* *p* < 0.05 versus non‐dialysis‐dependent CKD.

### 3.3 Association between AMH and vascular health measurements

The association between AMH and vascular health measurements, including measures of endothelial function (FMD, VTI) and arterial stiffness (AIx, PWV), stratified by dialysis status, is outlined in Table 3. In univariate analysis, no association was observed between AMH and each measure of vascular health. The initial multivariate model included covariates age, eGFR, ACR, and menstrual status, and examined a potential interaction effect between age and AMH, and did not demonstrate a statistically significant association between AMH and any measure of vascular health. After adjustment of the model through backward elimination, the final model utilized age as a covariate with an interaction effect between AMH and age. This model illustrated a significant association between AMH and FMD in the non‐dialysis‐dependent group, as well as AMH and AIx in all participants and the non‐dialysis‐dependent CKD group. No significant associations were apparent between AMH and any measure of vascular health in the dialysis‐dependent CKD group in any model.

**TABLE 3:** Association between AMH and vascular health parameters in women with chronic kidney disease

	Unadjusted model *R* ^2^ (*p*)	Multivariate adjusted model^a^ *R* ^2^ (*p*)	Multivariate adjusted model^b^ *R* ^2^ (*p*)	Multivariate adjusted model^c^ *R* ^2^ (*p*)	Multivariate adjusted model^d^ *R* ^2^ (*p*)
All participants					
AIX (%)	0.00 (0.71)	0.29 (0.06)	**0.32 (0.01)**	**0.31 (0.01)**	**0.18 (0.03)**
PWV (m/s)	0.01 (0.5)	0.21 (0.41)	0.10 (0.68)	0.07 (0.72)	0.04 (0.68)
FMD (%)	0.00 (0.9)	0.25 (0.13)	0.21 (0.11)	0.20 (0.07)	0.12 (0.11)
VTI (cm)	0.00 (0.6)	0.24 (0.15)	0.24 (0.06)	0.20 (0.08)	0.12 (0.12)
Non‐dialysis‐dependent CKD					
AIX (%)	0.01(0.62)	0.29 (0.06)	**0.29 (0.03)**	**0.29 (0.02)**	**0.20 (0.04)**
PWV (m/s)	0.02 (0.47)	0.21 (0.41)	0.16 (0.46)	0.09 (0.62)	0.07 (0.54)
FMD (%)	0.00 (0.92)	0.25 (0.13)	0.25 (0.08)	**0.24 (0.04)**	**0.21 (0.03)**
VTI (cm)	0.00 (0.98)	0.24 (0.15)	0.23 (0.10)	0.18 (0.14)	0.14 (0.15)
Dialysis‐dependent CKD					
AIX (%)	0.03 (0.73)	N/A	N/A	N/A	0.17 (0.88)
PWV (m/s)	0.02 (0.80)	N/A	N/A	N/A	0.88 (0.17)
FMD (%)	0.00 (0.90)	N/A	N/A	N/A	0.66 (0.29)
VTI (cm)	0.37 (0.15)	N/A	N/A	N/A	0.44 (0.57)

Abbreviations: ACR, albumin creatinine ratio; Aix, aortic augmentation index; AMH, anti‐Mullerian hormone; CKD, chronic kidney disease; eGFR, estimated glomerular filtration rate; FMD, flow‐mediated dilation; PWV, pulse wave velocity; VTI, velocity time integral.

^a^ Adjusted for age, ACR, menstruation, eGFR with interaction effect between age and AMH.

^b^ Adjusted for age, ACR, menstruation with interaction effect between age and AMH.

^c^ Adjusted for age and ACR with interaction effect between age and AMH.

^d^ Adjusted for age with interaction effect between age and AMH.

The bolded values reached statistical significance with *p* < 0.05.

## 4 DISCUSSION/CONCLUSION

To date, this is the first study to explore the relationship between measures of fertility and cardiovascular risk in young women with CKD. Key findings for young women with CKD were as follows: (1) lower AMH levels were associated with increased endothelial dysfunction, as measured by FMD, in non‐dialysis‐dependent CKD; (2) AMH levels were positively associated with AIx; and (3) AMH levels did not differ by CKD stage. These findings suggest that AMH and ovarian reserve may be important markers of future cardiovascular risk in young women with CKD.

CKD is considered the highest risk group for CVD mortality (GBD, 2017 Causes of Death Collaborators, 2018; Levey et al., 1998; Sarnak et al., 2002, 2003) and premenopausal‐aged women with CKD have a substantial CVD risk (Carrero et al., 2011; Nitsch et al., 2013). The risk relationship between cardiovascular mortality and CKD is amplified and CVD occurs earlier in the CKD disease course in women as compared to men (Nitsch et al., 2013). This elevated CVD risk in women with CKD is accentuated in premenopausal‐aged women, in which similar or increased cardiovascular mortality rates as compared to age‐matched men have been observed. These young women with CKD appear not to experience the sex‐related protective effect observed in premenopausal women in the general population or postmenopausal women with CKD (Carrero et al., 2011). Taken together, these observations highlight important potential for the contribution of female‐specific factors, such as ovarian reserve measured by AMH, in CKD‐related CVD risk in young women.

Female factor infertility in young women has recently been recognized as an important CVD risk factor (Hanson et al., 2017; Mahalingaiah et al., 2017; Quinn & Cedars, 2018). Low levels of anti‐Mullerian hormone (AMH), a surrogate marker of ovarian reserve, have been associated with CVD risk factors (obesity, insulin resistance, lipid disturbances, hypertension, and arterial intima‐medial thickness) (Bleil et al., 2013; van Dorp et al., 2013; Lambrinoudaki et al., 2020; Looby et al., 2016), as well as cardiovascular disease itself, notably coronary artery disease (de Kat et al., 2017). Although a causal relationship between AMH and CVD has not been fully established, upregulation of AMH receptor genes demonstrated in the hearts of neonates with hypoplastic left heart syndrome, suggests a systemic vascular role for AMH and supports the notion of a causal relationship between AMH and vascular health (Ricci et al., 2010). Additionally, the association noted between low levels of AMH and pre‐eclampsia, a disease of impaired vascular function, also may indicate a causative role of AMH in abnormal placental vascular development (Yarde et al., 2014). However, a non‐causative relationship between AMH and CVD is also possible in which factors such as inflammation may result in concurrent ovarian suppression and reduction of AMH, alongside increased CVD risk, which is supported by the observation of low AMH levels in many inflammatory chronic diseases (Wiles et al., 2021).

Female factor infertility is associated with CKD, and is thought to be increasingly prevalent through the stages of CKD, with those on dialysis at highest risk (Dumanski & Ahmed, 2019). Infertility in CKD appears to be driven by a derangement of female sex hormones, including AMH. Previous literature has clearly demonstrated that women with non‐dialysis‐dependent CKD have reduced AMH levels as compared to the general population (Sikora‐Grabka et al., 2016; Stoumpos et al., 2018; Wiles et al., 2021), even in early CKD stages (eGFR >60 ml/min/1.73 m^2^) (Wiles et al., 2021). Controversy exists with respect to AMH levels in the dialysis‐dependent CKD population, with early studies demonstrating higher AMH levels (Sikora‐Grabka et al., 2016; Stoumpos et al., 2018), but a more contemporary study suggesting lower AMH levels as compared to the general population (Wiles et al., 2021). In our study, the mean AMH level was 15.4 pmol/L, which was comparable to previous reports of AMH in the CKD population (6.33–23.6 pmol/L), and lower than the expected AMH levels in the general population (Klemt et al., 2020; Wiles et al., 2021). Furthermore, a preserved relationship between AMH and age was maintained in our CKD population. Interestingly, no difference in AMH levels was observed between non‐dialysis‐dependent and dialysis‐dependent CKD groups, which was in keeping with previous observations (Wiles et al., 2021). Moreover, there was no apparent association between AMH and eGFR, and AMH levels did not differ between CKD stages in the non‐dialysis‐dependent CKD population. This observation highlights that although the AMH levels in our study population were consistently lower than previously reported levels in the general population, the levels did not appear to be linked or change based on kidney function.

In this study, a significant association was demonstrated between AMH and FMD in the population of premenopausal‐aged women with CKD. It is well established that macrovascular endothelial function, as measured by FMD, is predictive of future cardiovascular morbidity and mortality in the general population (Martin & Anderson, 2009). There is increasing evidence that endothelial function is also predictive of cardiovascular morbidity and mortality in CKD; and FMD has been linked to increased inflammation and carotid artery intimal medial thickness (Recio‐Mayoral et al., 2011; Yilmaz et al., 2011), increased left ventricular (LV) mass (Dubin et al., 2016), and increased major adverse cardiovascular events (Lee et al., 2014) in the CKD population. Our data demonstrated increased vascular dysfunction in women with lower levels of AMH and provides a novel observation that AMH may be an independent predictor of endothelial dysfunction and future cardiovascular risk in the young female population with CKD.

The data also demonstrated a significant relationship between AMH and AIx, but not PWV, in premenopausal‐aged women with CKD. PWV is considered the gold standard measurement for arterial stiffness, and imperfect correlation exists between PWV and AIx (Laurent et al., 2006; Sakurai et al., 2007). AIx is a dynamic and composite measure of vascular response, and represents not only arterial stiffness, but also other factors such as endothelial function and peripheral vascular tone. It is possible that the relationship we detected between AMH and AIx in this study represents a more composite reflection of vascular function, and the nature of the relationship between AMH and AIx in the premenopausal‐aged CKD population remains unclear.

Despite a relationship between AMH and vascular dysfunction in the non‐dialysis‐dependent CKD population, no relationship between AMH and vascular health was identified in the dialysis‐dependent CKD population, which may reflect our limited sample size. Previous studies have demonstrated that baseline vascular health differs between non‐dialysis‐dependent CKD and dialysis‐dependent CKD populations, and that vascular dysfunction tends to increase alongside severity of CKD (Wang et al., 2005). With the exception of AIx, we did not find any significant differences between the two CKD groups with respect to baseline vascular health measurements. It is possible that our small sample size (*N* = 7) was not reflective of the general female dialysis‐dependent CKD population. Alternatively, it is well established that those with dialysis‐dependent CKD have a significantly higher risk of CVD compared to the non‐dialysis‐dependent CKD population (GBD, 2017 Causes of Death Collaborators, 2018; Sarnak et al., 2002), and have a multitude of unique factors (some known, others unknown) that influence their vascular health (Cuenca et al., 2020; Wang et al., 2005). Therefore, it is possible that the relationship between AMH and vascular dysfunction demonstrated in the non‐dialysis‐dependent CKD population becomes muted or blunted in the context of other overwhelming factors specific to vascular health in those with dialysis‐dependent CKD.

This study had several strengths. The study cohort was diverse in terms of both age, ethnicity, and etiology of CKD. We were able to recruit participants with a wide range of eGFR, as well as treated with both hemodialysis and peritoneal dialysis. Our study protocol was standardized to each participant's menstrual cycle (when applicable), and is well established and previously described. Additionally, all arterial stiffness and endothelial function measurements were completed by consistent research personnel. This study also had important limitations. First, although it is the largest study of its kind to date, the sample size was relatively small, limiting the power of the study. Furthermore, the dialysis‐dependent CKD group had only seven participants, which restricted our ability to draw firm conclusions from this group. Additionally, despite the overall diversity demonstrated in our CKD study population, there was limited representation of late stage non‐dialysis‐dependent CKD, which limits our generalizability of study findings to this group. Another important limitation of our study was the absence of a healthy control group to allow for comparisons between the CKD population and the general population, though our findings of vascular dysfunction in the CKD population are consistent with previous reports (Dubin et al., 2016; Lee et al., 2014; Martin & Anderson, 2009; Recio‐Mayoral et al., 2011; Yilmaz et al., 2011). Finally, to minimize potential for residual confounding, we controlled for important factors known to impact vascular health in this population. We were unable to examine the effects of smoking status, coexisting inflammatory diseases, or gendered variables.

In summary, we demonstrated that lower AMH levels are associated with vascular dysfunction, a validated predictor of future CVD, in premenopausal‐aged women with CKD. The relationship illustrated between AMH and vascular dysfunction in the young female CKD population may provide insight into CVD risk factors unique to young women. Larger studies are urgently needed to further investigate the relationship between measures of ovarian reserve and CVD in women.

## CONFLICT OF INTEREST

None declared.

## AUTHOR CONTRIBUTIONS

Study conception: Sandra M. Dumanski and Sofia B. Ahmed; Study design: Sandra M. Dumanski, Todd J. Anderson, Kara A. Nerenberg, Jayna Holroyd‐Leduc, Jennifer MacRae, Amy Metcalfe, Satish R. Raj, Sharanya Ramesh, Cindy Z. Kalenga, and Sofia B. Ahmed; Data acquisition: Sandra M. Dumanski, Todd J. Anderson, Darlene Sola, Milada Pajevic, and Sofia B. Ahmed; Data analysis and interpretation: Sandra M. Dumanski, Todd J. Anderson, Kara A. Nerenberg, Jayna Holroyd‐Leduc, Satish R. Raj, and Sofia B. Ahmed; manuscript draft and revision: Sandra M. Dumanski, Todd J. Anderson, Kara A. Nerenberg, Jayna Holroyd‐Leduc, Jennifer MacRae, Amy Metcalfe, Satish R. Raj, Sharanya Ramesh, Cindy Z. Kalenga, Darlene Sola, Milada Pajevic, and Sofia B. Ahmed.
